# Platelet and Monocyte Activation After Transcatheter Aortic Valve Replacement (POTENT-TAVR): A Mechanistic Randomized Trial of Ticagrelor Versus Clopidogrel

**DOI:** 10.1016/j.shj.2023.100182

**Published:** 2023-04-28

**Authors:** David A. Zidar, Sadeer Al-Kindi, Chris T. Longenecker, Sahil A. Parikh, Carl B. Gillombardo, Nicholas T. Funderburg, Steven Juchnowski, Lauren Huntington, Trevor Jenkins, Christopher Nmai, Michael Osnard, Mehdi Shishebhor, Steven Filby, Curtis Tatsuoka, Michael M. Lederman, Eugene Blackstone, Guilherme Attizzani, Daniel I. Simon

**Affiliations:** a Department of Medicine, Case Western Reserve University, Cleveland, Ohio, USA; b Louis Stokes Cleveland Veterans Affairs Medical Center, Cleveland, Ohio, USA; c Harrington Heart & Vascular Institute, University Hospitals Cleveland Medical Center, Cleveland, Ohio, USA; d Division of Cardiology, Center for Interventional Vascular Therapy, Columbia University Irving Medical Center, New York, New York, USA; e Division of Medical Laboratory Science, School of Health and Rehabilitations Sciences, Ohio State University, Columbus, Ohio, USA; f New York University Grossman School of Medicine, New York, New York, USA; g Department of Population Health and Quantitative Health Sciences, Cleveland Clinic Foundation, Cleveland, Ohio, USA

**Keywords:** Clopidogrel, Inflammation, TAVR, Ticagrelor, Transcatheter aortic valve replacement

## Abstract

**Background:**

Inflammation and thrombosis are often linked mechanistically and are associated with adverse events after transcatheter aortic valve replacement (TAVR). High residual platelet reactivity (HRPR) is especially common when clopidogrel is used in this setting, but its relevance to immune activation is unknown. We sought to determine whether residual activity at the purinergic receptor P2Y12 (P2Y12) promotes prothrombotic immune activation in the setting of TAVR.

**Methods:**

This was a randomized trial of 60 patients (enrolled July 2015 through December 2018) assigned to clopidogrel (300mg load, 75mg daily) or ticagrelor (180mg load, 90 mg twice daily) before and for 30 days following TAVR. Co-primary endpoints were P2Y12-dependent platelet activity (Platelet Reactivity Units; VerifyNow) and the proportion of inflammatory (cluster of differentiation [CD] 14+/CD16+) monocytes 1 day after TAVR.

**Results:**

Compared to clopidogrel, those randomized to ticagrelor had greater platelet inhibition (median Platelet Reactivity Unit [interquartile range]: (234 [170.0-282.3] vs. 128.5 [86.5-156.5], *p* < 0.001), but similar inflammatory monocyte proportions (22.2% [18.0%-30.2%] vs. 25.1% [22.1%-31.0%], *p* = 0.201) 1 day after TAVR. Circulating monocyte-platelet aggregates, soluble CD14 levels, interleukin 6 and 8 levels, and D-dimers were also similar across treatment groups. HRPR was observed in 63% of the clopidogrel arm and was associated with higher inflammatory monocyte proportions. Major bleeding events, pacemaker placement, and mortality did not differ by treatment assignment.

**Conclusions:**

Residual P2Y12 activity after TAVR is common in those treated with clopidogrel but ticagrelor does not significantly alter biomarkers of prothrombotic immune activation. HRPR appears to be an indicator (not a cause) of innate immune activation in this setting.

## Graphical abstract

The Platelet and Monocyte Activation After Transcatheter Aortic Valve Replacement trial was a randomized trial of clopidogrel vs. ticagrelor to test the hypothesis that high HRPR despite clopidogrel use after transcatheter aortic valve replacement (TAVR) contributes to postprocedural monocyte activation and elevated biomarkers of immuno-thrombosis. We find that those who were clopidogrel-responsive had lower levels of inflammatory monocytes 1 day after TAVR (Kruskal-Wallis test, Dunn’s posttest, ∗∗: *p* < 0.01), compared to those with HRPR on clopidogrel or those randomized to ticagrelor. However, ticagrelor did not lead to reductions in immunologic or thrombotic biomarkers, compared to clopidogrel. Thus, residual P2Y12 responsiveness does not appear to promote immune activation after TAVR; instead, clopidogrel metabolism or efficacy may be diminished as consequence or epiphenomenon of robust inflammation in the post-TAVR setting.

**Figure:**
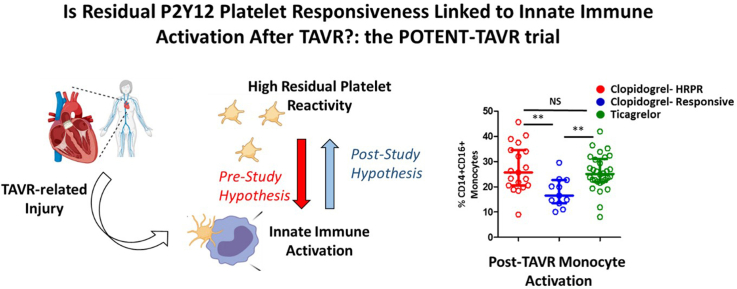

## Introduction

Transcatheter aortic valve replacement (TAVR) has emerged as an important alternative to surgical aortic valve replacement.^1^ In hospital mortality is low (<3%), and valve function is generally durable beyond 5 years. However, excess mortality (relative to comorbidity equivalent patients without aortic stenosis) exists for an additional 2 ​years postprocedure.^2^ The absolute risk accrued during this postacute period is substantial: 2-year mortality rates approach 15%^3^^,^^4^ and 20% to 35%^5^^,^^6^ for those at intermediate and high surgical risk, respectively. Understanding the modifiable factors that promote adverse clinical events in this intermediate time frame may allow for further improvements in post-TAVR outcomes.

Thrombotic events after TAVR include the risk of stroke,^7^ myocardial infarction,^8^ and hypoattenuated valve leaflet thickening which may predispose to valve thrombosis or accelerated prosthesis failure.^9^, ^10^, ^11^, ^12^ Heart failure and sepsis are the more common causes of intermediate term morbidity and mortality,^13^ and a body of evidence links myocardial recovery after TAVR to periprocedural immune dysfunction.^14^, ^15^, ^16^, ^17^ However, the mechanisms that contribute to thrombosis and/or immune dysfunction and their causal position relative to clinical events have yet to be defined.

Activated platelets are positioned proximally in many inflammatory responses via direct and indirect interactions with leukocytes. P2Y12 inhibition has been shown to protect against thrombotic events and death in acute coronary syndromes,^18^ and may also improve survival in the setting of pneumonia and sepsis.^19^, ^20^, ^21^ TAVR potentially initiates multiple simultaneous exposures (native valve disruption, hemodynamic insults, bioprosthesis antigenicity, vascular injury, etc.) which could trigger platelet activation, platelet-monocyte aggregation, innate immune activation, proinflammatory cytokine expression, and stimulation of coagulation/fibrinolytic pathways. Compared to classical (CD14+/CD16−) monocytes, the CD14+/CD16+ “inflammatory/intermediate” subset are more prone to bind activated platelets,^22^ express higher levels of tissue factor,^23^ and secrete proinflammatory cytokines.^24^ The proportion of inflammatory monocytes to total monocytes is among the strongest immune biomarker linked prospectively to adverse clinical events,^25^ including recovery after TAVR.^15^

In the early years after the FDA approval of TAVR, dual antiplatelet therapy (DAPT) with clopidogrel and aspirin was often prescribed empirically for 3 to 6 months after TAVR, consistent with European and the United States guidelines.^26^, ^27^, ^28^ Several groups have reported exceedingly high rates (∼70%) of high residual platelet reactivity (HRPR) when clopidogrel is used in this setting,^29^^,^^30^ and therefore whether the expected salutary immunothrombotic effects of P2Y12 inhibition are achieved with clopidogrel in this setting are uncertain. The Platelet and Monocyte Activation After Transcatheter Aortic Valve Replacement (POTENT-TAVR) trial was undertaken to address this knowledge gap. Recent studies find that the addition of clopidogrel to aspirin does not reduce thrombotic events after TAVR, and guidelines have generally downgraded the need for “DAPT” due to the increased risk of non-fatal bleeding.^31^, ^32^, ^33^, ^34^ However, the extent to which post-TAVR immunothrombotic activation is truly independent of P2Y12 vs. inadequately suppressed by clopidogrel remains unknown, relevant to the interpretation of completed trials, and instructive to the development of immunothrombotics with lower bleeding risk.

**Figure 1:**
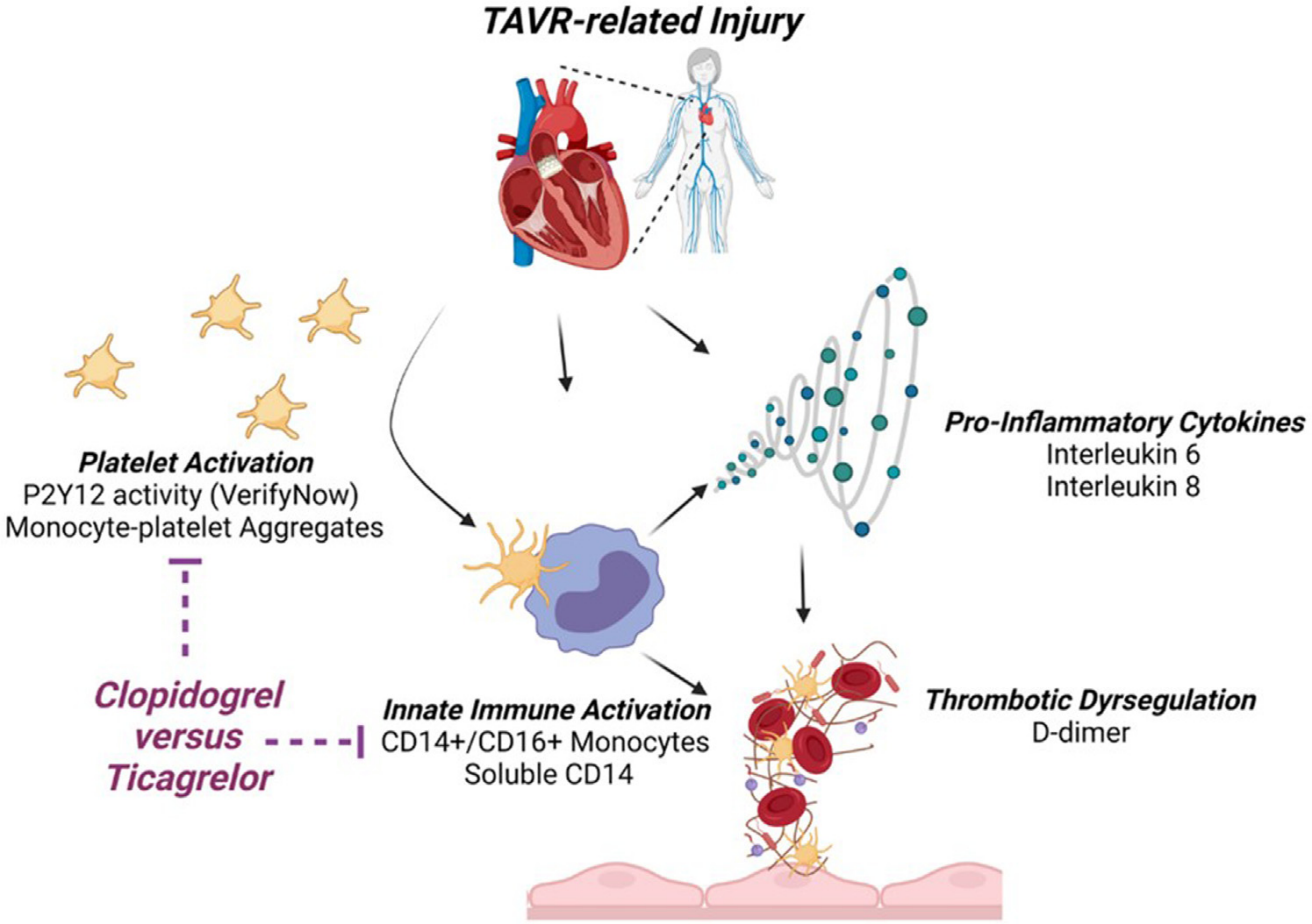
**A randomized trial to characterizeimmune and thrombotic activation after transcatheter aortic valve replacement (TAVR).** The Platelet and Monocyte Activation After Transcatheter Aortic Valve Replacement trial is a randomized trial of clopidogrel vs. ticagrelor to test whether the potency of P2Y12 inhibition influences innate immune activation, inflammatory cytokine elevations, and thrombotic dysregulation after TAVR.

Here, we report the completed biomarker analysis of the POTENT-TAVR trial. Enrollment was from July 2015 through December 2018, and patients without an indication for anticoagulation were randomized to clopidogrel vs. ticagrelor in a background of low dose aspirin. We sought to test the hypothesis that, compared to clopidogrel, ticagrelor would result in higher potency P2Y12 inhibition *and* greater reductions in inflammatory monocytes in the post-TAVR setting (Figure 1). We find that although ticagrelor results in demonstrably greater platelet inhibition, clopidogrel was sufficient (i.e., similar to ticagrelor) for suppression of P2Y12-dependent immunothrombosis.

## Methods

### Trial Design

The POTENT-TAVR trial was a prospective, randomized, open-label investigator-initiated trial of patients with severe symptomatic degenerative aortic stenosis undergoing planned transfemoral TAVR. The exclusion criteria were prior history of stroke, transient ischemic attack, intracranial hemorrhage, an established bleeding diathesis, thrombocytopenia (<150 k/uL), recent (<12 months) or active excessive bleeding, current infection, history of autoimmune disease, established allergy to contrast agents, thienopyridines, aspirin or ticagrelor, atrial fibrillation, deep venous thrombosis, pulmonary embolism, or other indication for long term anticoagulation. Full details of the design are provided as a Supplementary Study Protocol.

Patients were randomized in a 1:1 ratio. Patients assigned to clopidogrel were treated with a 300 mg load on the morning of TAVR, and 75 mg daily after TAVR for a total of 30 days. Those assigned to ticagrelor received a 180 mg loading dose, followed by 90 mg twice daily for 30 days. All patients received aspirin 81 mg daily (after a 325 mg loading dose if aspirin naïve).

The TAVR procedure was performed according to local standards, using a minimalist approach consisting of conscious sedation and percutaneous access without surgical cutdown.^35^ Patients received intravenous heparin (∼100 U/kg), with additional doses administered if the activated clotting time was below 250 ​seconds. Patients were typically discharged within 24 to 48 ​hours in the absence of complications. Postdischarge compliance with study drug and adverse events was assessed by the study staff in hospital and in conjunction with outpatient clinical visits at day 7 and day 30 post-TAVR. Bleeding was assessed according to the Bleeding Academic Research Consortium and Valve Academic Research Consortium-2 classifications.^36^ The CONSORT diagram describing the details of enrollment and participation is included as Supplemental Figure 1.

### Ethical Considerations

The study was approved by the institutional research board at University Hospitals Cleveland Medical Center, and complied with the Declaration of Helsinki, the International Conference on Harmonization/Good Clinical Practice guidelines, and applicable local regulatory requirements. All patients provided informed consent prior to enrollment. The trial was registered at ClinicalTrials.gov (Identifier: NCT02486367). The protocol was granted an investigational new drug waiver from the United States Federal Drug Administration prior to study initiation. A data safety and monitoring board was appointed and met at quarterly enrollment intervals to review adverse events throughout the trial period. The study was an investigator-initiated study funded by AstraZeneca (Co-Principal Investigators: D.A.Z. and D.I.S.). The funders had no role in study design, data collection and analysis, decision to publish, or preparation of the manuscript.

### Study Endpoints and Variables

Platelet reactivity to adenosine diphosphate was assessed as a primary endpoint and reported as platelet reactivity units (PRU) using the VerifyNow P2Y12 assay (Accriva Diagnostics, San Diego, California).^37^ The second primary endpoint was the percentage of CD14+CD16+ monocytes relative to total monocytes, measured by flow cytometry as previously described^23^ and as shown in Supplemental Figure 2. These dual primary endpoints were assessed at 1 day after TAVR.

Pre-specified secondary endpoints include platelet reactivity and inflammatory monocytes at day 7 and day 30 after TAVR, monocyte-platelet aggregates (MPAs, measured by flow cytometry using antibodies to CD62P (P-selectin) or activated IIb/IIIa as shown in Supplemental Figure 3), interleukin (IL)-6 (measured using enzyme linked immunosorbent assays, ELISA; R and D systems), D dimers (ELISA; Abcam), and soluble CD14 (soluble CD14 [sCD14]; ELISA; R and D systems). HRPR was defined as a PRU >208 in accordance with consensus recommendations and previous studies.^30^^,^^38^

Clinical data elements were defined according to the Transcatheter Valve Therapy registry definitions.^39^ Society of Thoracic Surgeons surgical risk scores were calculated for 30 day postsurgical mortality assuming isolated aortic valve replacement using model version 2.81 (http://riskcalc.sts.org/stswebriskcalc).^40^ Major bleeding events were defined as BARC type 3a or greater. All-cause mortality was identified via clinic followup and linkage with Ohio state death records.

### Statistical Methods

Analyses were conducted according to an intention to treat. The sample size was determined based upon preliminary data in 25 patients in which the mean proportion of inflammatory monocytes 1 day after TAVR was 20.0% with a standard deviation of 5.0%, and thus our sample size of 60 total patients was chosen to provide 80% power to detect an absolute difference of 4% for the inflammatory monocyte endpoint, assuming type 1 error of 0.025 given Bonferroni correction to account for our 2 dual primary endpoints. No adjustment for multiple comparisons was performed for secondary comparisons. Missingness was assumed to be completely at random and expected to be rare since the primary endpoints would be obtained prior to discharge. Categorical variables are expressed as percentages and were compared using Fisher’s exact tests. Continuous variables are expressed as means (SD) and median (interquartile range, IQR). Primary and secondary biomarker outcomes were analyzed using Mann-Whitney *U* tests. Linear mixed models were used to analyze the PRU and Inflammatory monocytes relative to treatment, time, and time by treatment interaction. Similarly, linear mixed models were used to characterize HRPR. Spearman correlations were performed to describe and analyze relationships between single biomarkers. To understand potential multivariable relationships among biomarkers, biomarker values from days 0, 1, 7, and 30 were converted into standardized values and principal components analysis performed using Varimax rotation with Kaiser Normalization to extract components with eigenvalues greater than 1. Logistic regression was used to identify predictors of HRPR and bleeding. Time to event (all-cause mortality) was analyzed by Kaplan-Meier curves and Mantel Cox log rank test. All analyses were performed using Statistical Package for Social Sciences (SPSS) (IBM Corporation, Armonk, New York) version 26. Art was created using Biorender.

## Results

### Study Population

The baseline clinical features of the study population (n = 60) are shown in Table 1. The median age is 86 years (IQR: 82-90), 34 (56.7%) identify as female, 3 (5%) are non-White. The median aortic valve area was 0.7 (IQR: 0.63-0.84) cm^2^. Prior to study drug initiation, 44/60 patients were on aspirin, 16/60 were on clopidogrel, and 2/60 were on ticagrelor. A Medtronic Corevalve (Medtronic Minneapolis, MN) was used in 46 (76.7%) patients and Edwards Lifesciences Sapien XT (Edwards Lifesciences Irvine, CA) was used in 14 (23.3%). Cardiovascular risk factors and comorbid illnesses were generally similar by treatment assignment.

**Table 1:** Baseline clinical characteristics

Variable	All patients (n = 60)	Clopidogrel (n = 30)	Ticagrelor (n = 30)	*p* value
Demographic/clinical characteristics				
Age	86 (82, 90)	86 (80, 90)	85 (82, 89)	0.994
Female sex (%)	34 (56.7%)	14	20	0.192
Non-Caucasian (%)	3 (5%)	2	1	1
HTN	52 (87%)	25	27	0.706
DM	18 (30%)	9	9	1
DL	37 (61.7%)	16	21	0.288
Current tobacco use	2 (3.3%)	2	0	0.492
Prior tobacco use	30 (50%)	14	16	0.797
COPD	11 (18.3%)	6	5	1
PAD	7 (11.7%)	2	5	0.424
Prior malignancy	15 (25%)	6	9	0.552
Prior CAD	31 (51.7%)	14	17	0.606
STS score	4.3 (3.0, 5.5)	3.9 (3.1, 5.6)	4.6 (3.0, 5.5)	0.197
HAS-BLED score	2 (2, 2)	2 (2, 2)	2 (2, 2)	0.177
Procedural characteristics				
CoreValve/Evolut R	46 (77%)	23	23	1
Sapien XT	14 (23%)	7	7	1
General anesthesia	0	0	0	1
Transfemoral access	60	60	60	1
Surgical femoral cutdown	0	0	0	1
Fluoroscopy time	19.7 (17.2, 27.1)	19.6 (17.1, 26.5)	20 (17.2, 29.0)	0.435
Contrast volume	68.0 (42.0, 83.0)	66 (44.1, 80.0)	77.0 (41, 96.5)	0.564
Aortic valve area	0.70 (0.63, 0.84)	0.75 (0.64, 0.86)	0.7 (0.58, 0.82)	0.605
Mean aortic alve gradient	40 (31, 48)	40 (28, 51)	39 (33, 46)	0.796
Left ventricular ejection fraction	60 (50, 65)	58 (45, 65)	65 (55, 65)	0.038
Baseline medications				
ACE inhibitor	12 (20%)	5	7	0.748
ARB	12 (20%)	4	8	0.333
BB	38 (63.3%)	19	19	1
Aspirin	44 (73.3%)	20	24	0.382
Clopidogrel	16 (26.7%)	8	8	1
Ticagrelor	2 (3.3%)	0	2	0.589
Statin	39 (65%)	21	18	0.417
Diuretics	32 (53.3%)	16	16	1
Insulin	8 (13.3%)	3	5	0.706
Oral hypoglycemics	9 (15%)	5	4	1
Antibiotics	1 (1.7%)	0	1	1
Chronic corticosteroid use	6 (10%)	3	3	1
Baseline laboratory measures				
Baseline creatinine (mg/dL)	1.01 (0.8, 1.2)	1.03 (0.75, 1.32)	0.96 (0.8, 1.2)	0.624
Baseline creatinine clearance (mL/min)	46.05 (29.5, 67.2)	48.5 (28.9, 72)	45 (31, 66)	0.762
Baseline WBC	7.2 (5.8, 8.1)	7.2 (5.8, 8.2)	7.25 (5.8, 8.0)	0.912
Baseline Hb (g/dL)	11.9 (10.7, 13.1)	11.9 (10.5, 13.5)	12 (10.7, 12.9)	0.762
Baseline PLT	217 (177.5270.8)	213.5 (175.2, 270.2)	220.5 (180, 275.5)	0.701
Baseline RDW	13.6 (13.1, 14.3)	13.7 (13.2, 14.3)	13.6 (12.9, 14.5)	0.611

*p* values reflect Fisher's Exact Test (discrete variables) or Mann-Whitney U tests (continuous variables) comparing clopidogrel vs. ticagrelor groups. Continuous variables are reported as median (interquartile range).

Abbreviations: ACE, angiotensin converting enzyme inhibitor; ARB, angiotensin receptor blocker; BB, beta blocker; Hb, hemoglobin (Hb); PLT, platelet count (PLT); RDW, red cell distribution width (RDW); WBC, white blood cell count; CAD, coronary artery disease; COPD, chronic obstructive pulmonary disease; DL, dyslipidemia; DM, diabetes mellitus; HAS-BLED, Bleeding Risk Score; HTN, hypertension; PAD, peripheral arterial disease; STS, Society of Thoracic Surgeons.

During the 30 day treatment period, there were no strokes or deaths. Ten patients experienced a major (BARC 3a or above) bleeding event within 30 days after TAVR, 8 of which occurred during the inpatient course and 2 were delayed (post-discharge). Five bleeds (1 delayed) occurred among the clopidogrel group, and 5 were among ticagrelor treated participants (1 delayed). Predictors of major bleeding included several inflammatory indices as shown in Supplemental Table 1. Permanent pacemaker placement was required in 6/30 and 3/30 patients assigned to clopidogrel and ticagrelor, respectively (*p* = 0.472). All cause survival after TAVR was similar between treatment groups and was 83.3% at 1 year in both arms (Supplemental Figure 4).

### Platelet Reactivity and Inflammatory Monocyte Activation After TAVR (Primary Endpoints)

**Figure 2:**
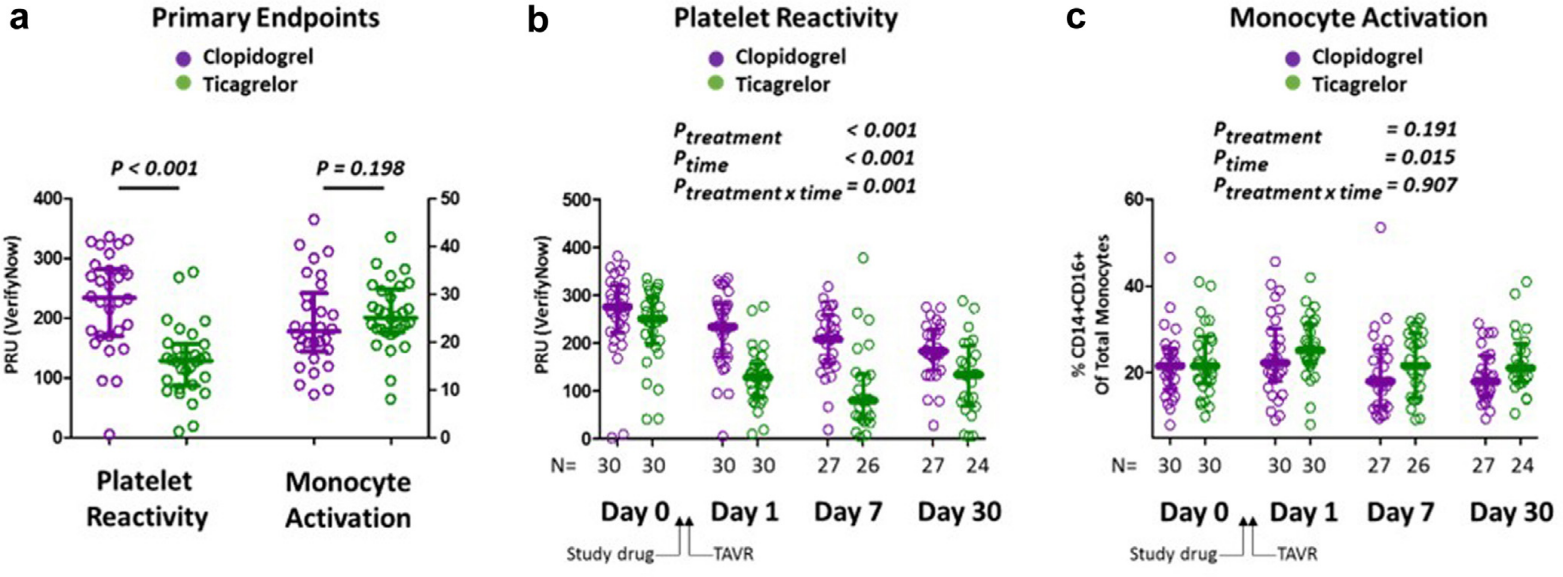
**Platelet reactivity and monocyte activation after TAVR according to P2Y12 inhibitor treatment assignment.** (a) Platelet reactivity (left axis) and monocyte activation (right axis) are shown for patients assigned to receive Clopidogrel (purple circles) or Ticagrelor (green circles) (Bars: median/interquartile range; *p* values: Mann-Whitney *U* tests). The time course of P2Y12 specific platelet reactivity (b) and the proportion of inflammatory (CD14+/CD16+) monocytes (c) are shown according to treatment assignment for days 0 (pre-TAVR, prestudy drug), 1 (1 day post-TAVR), 7, and 30 after TAVR (*p* values: linear mixed models for treatment, time, and treatment by time interaction). Abbreviations: PRU, platelet reactivity unit; TAVR, transcatheter aortic valve replacement.

The primary endpoints of platelet reactivity and monocyte activation in the acute post-TAVR setting are shown in Figure 2B and C. The median (IQR) PRU 1 day post-TAVR was significantly lower in the ticagrelor arm than the clopidogrel arm (ticagrelor median 128.5 [IQR: 86.5-156.5] vs. clopidogrel median 234 [IQR: 170.0-282.3]; Mann-Whitney *U* test: *p* < 0.001). The proportion of inflammatory monocytes 1 day after TAVR was similar between those receiving ticagrelor and clopidogrel (ticagrelor median 25.1 [IQR: 22.1-31.0] vs. clopidogrel median 22.2 [IQR: 18.0-30.2]; Mann-Whitney *U* test: *p* = 0.198).

Differences in platelet reactivity and similarity in monocyte activation were generally maintained throughout the 30-day study period (Figure 1B and C). Linear mixed models suggest the effect of treatment and the treatment by time interaction was significant for platelet reactivity (*p* = 0.001), but not for inflammatory monocyte proportions (*p* = 0.907).

### Secondary Markers of Immuno-Thrombotic Risk

**Figure 3:**
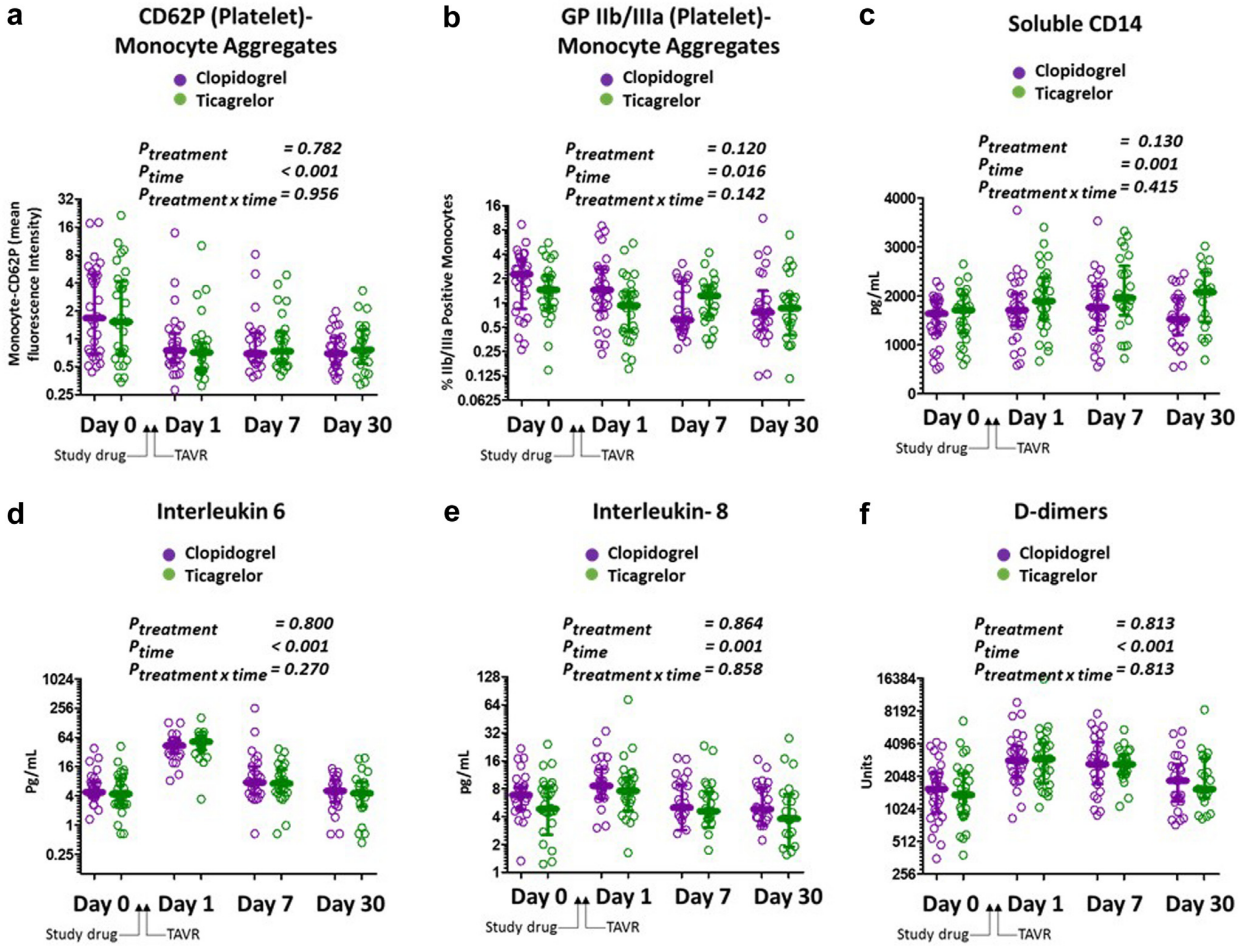
**Secondary inflammatory and thrombotic measures according to P2Y12 inhibitor treatment assignment.** The time course of (a) CD62P (platelet)/monocyte aggregates (mean fluorescence intensity of P-selectin on monocytes), (b) glycoprotein IIb/IIIa/monocyte aggregates, (c) soluble CD14 levels, (d) plasma interleukin 6 concentrations, (e) interleukin 8 concentrations, (f) and plasma D-dimer levels are shown on day 0 (pre-TAVR, prestudy drug), day 1 (1 day post-TAVR), day 7, and 30 days after TAVR for patients assigned to receive clopidogrel (purple circles) or ticagrelor (green circles). (Bars: median/interquartile range; *p* values: linear mixed models for treatment, time, and treatment by time interaction). Abbreviation: TAVR, transcatheter aortic valve replacement.

The percentage of circulating MPAs as measured using CD62P and activated glycoprotein IIb/IIIa, plasma levels of soluble CD14 (sCD14) levels, interleukin 6 (IL-6), plasma interleukin 8 (IL-8) levels, and D-dimer levels are shown in Figure 3 and Supplemental Table 2. MPA tended to decline after study drug initiation and TAVR (p_time_ < 0.001 for CD62P + MPA and 0.016 for IIb/IIIa + MPA) whereas sCD14, IL6, IL8, and D-dimers increased (p_time_ for time ≤0.001 for each). The percentage of MPA, and levels of sCD14, IL-6, IL-8, and D-dimers were similar in both treatment arms (p_treatment by time interaction_ > 0.05 for each).

Inflammatory monocyte proportions tended to correlate with CD62P MPA at baseline (Spearman rho = 0.310, *p* = 0.016; Supplemental Figure 5A), but this relationship was attenuated after study drug initiation/TAVR. SCD14 was associated with inflammatory monocyte proportions and IL-6 at day 0 but more closely related to inflammatory monocyte at day 1. D-dimers, IL-6, and IL-8 tended to be closely related to each other, especially after study drug initiation/TAVR. Principal components analysis of biomarker measures was also consistent with a complex but highly compartmentalized immune-thrombotic milieu (Supplemental Figure 5B). PRU, monocyte-CD62P MPA, and monocyte-IIb/IIIa MPA tended to vary as a unit (component 2) which was distinct from the variance of IL-6, IL-8, and D-dimer levels (component 1). Inflammatory monocyte proportions co-varied with sCD14 levels and to a lesser extent MPA and IL-6 (component 3).

### High Residual Platelet Reactivity After TAVR

**Figure 4:**
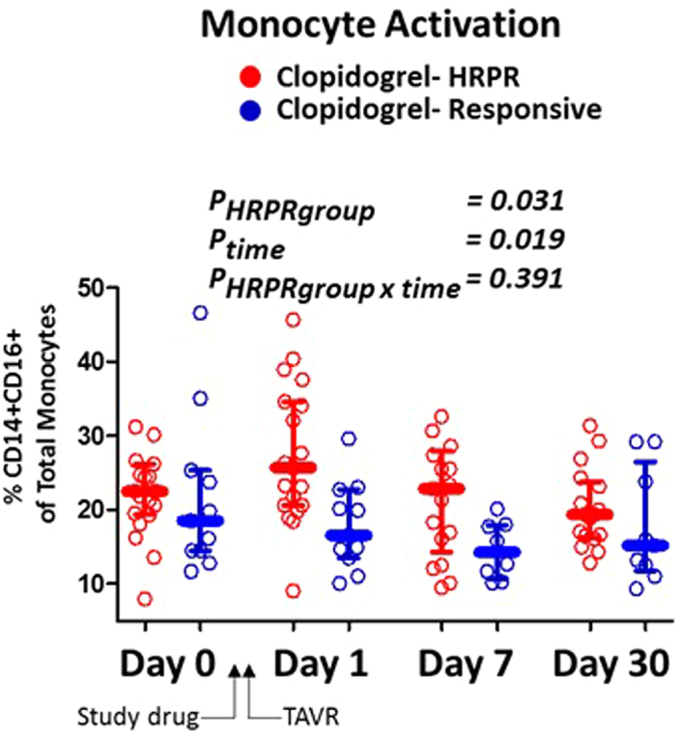
**Clopidogrel responsiveness is associated with diminished inflammatory monocyte expansion.** The proportion of inflammatory (CD14+/CD16+) monocytes among those assigned to clopidogrel who experienced high residual platelet reactivity (HRPR, platelet reactivity unitsPRU > 208; red) at day 1 are compared to those assigned to clopidogrel who were responsive (platelet reactivity unit ≤208; blue). (Bars: median/interquartile range; *p* values: linear mixed models for HPRR status, time, and HRPR status by time interaction). Abbreviations: HRPR, high residual platelet reactivity; TAVR, transcatheter aortic valve replacement.

Among those randomized to clopidogrel, the proportion who had HRPR (PRU >208) at days 1, 7, and 30 was 63% (n = 19/30), 52% (n = 14/27), and 37% (n = 10/27). Thus, although HRPR was common acutely after TAVR, rates tended to decline during the 30 day treatment period. HRPR was not significantly associated with individual clinical/demographic factors (Supplemental Table 3), but those with HRPR tended to have higher inflammatory monocyte proportions compared to those who were clopidogrel-responsive (Supplemental Table 3 and Figure 4).

## Discussion

We performed a prospective, randomized biomarker trial to test whether higher potency P2Y12 inhibition with ticagrelor, compared to clopidogrel, provides greater platelet inhibition and suppression of prothrombotic immune activation in the setting of TAVR. We found that ticagrelor led to demonstrably greater platelet P2Y12 inhibition compared to clopidogrel in the post-TAVR setting. However, ticagrelor did not reduce the proportion of inflammatory monocytes or plasma levels of sCD14, IL-6, IL-8, or D-dimers compared to clopidogrel. We conclude that residual P2Y12 activity does not contribute to heterogeneity in the activation of monocytes or selected markers of prothrombotic inflammation after TAVR. By extension, the degree of platelet inhibition afforded by clopidogrel appears to be sufficient for the suppression of P2Y12-dependent immunothrombosis in this setting.

### HRPR in the peri-TAVR setting is common despite Clopidogrel, associated with immune activation, and can be circumvented with Ticagrelor

Clopidogrel is a prodrug that requires the cytochrome P450 system for conversion to the active P2Y12 inhibiting metabolite. HRPR can reflect genetic polymorphism at cytochrome P450 2C19 (CYP2C19), but more commonly results from acquired alterations in CYP2C19 expression, possibly related to age, obesity, diabetes, left ventricular dysfunction, concurrent medications, or inflammation.^41^ P2Y12 receptor expression and levels of G-protein receptor kinases may also account for heterogeneity of drug effects.^42^ HRPR is among the strongest predictors of major adverse cardiovascular events in those with indications for DAPT.^41^ In contrast to clopidogrel, ticagrelor does not require hepatic metabolism, binds the receptor reversibly, and results in higher potency inhibition of P2Y12.^43^

The results of this study demonstrate that HRPR is indeed exceedingly common when clopidogrel is used in the peri-TAVR setting. The rates we observed (63.3%) are comparable to the 68% rate reported in our pilot study^29^ and 71% reported in the Assessment of platelet REACtivity after Transcatheter Aortic Valve Implantation (REAC-TAVI) trial.^30^ Interestingly, heightened inflammatory monocyte proportions are the major predictor of HRPR in this study. A potential association between HRPR and inflammation has been postulated previously. For instance, certain inflammatory signals diminish hepatic CYP2C19 expression,^44^ which could reduce conversion of clopidogrel to its active metabolite. Proinflammatory cytokines are also known to impart major effects on gene expression in megakaryocytes,^45^ but whether these pathways alter P2Y12 regulation and signaling is unknown. On-clopidogrel PRUs tended to decline during the post-TAVR follow-up period which could be consistent with transient pharmacokinetic or inflammatory processes as a basis of HRPR in this setting.

### Inflammatory monocyte expansion after TAVR is not related to the potency of platelet P2Y12 inhibition

The mechanisms linking inflammation and thrombosis are multiple and broadly relevant to infectious/inflammatory conditions, cancer, cardiovascular disease, and other adverse clinical events. In highly inflammatory settings (e.g., pneumonia, sepsis, HIV), markers of platelet activation, innate immune activation, and thrombophilia often associate with each other. For instance, inflammatory monocyte proportions, CD62P, sCD14, IL-6, and D-dimers strongly correlate with each other in HIV disease.^46^ In pneumonia, platelet activation may be upstream and contribute to inflammation since ticagrelor reduces IL-6 levels and mortality in this setting.^19^^,^^47^

Here, the lack of reduction in the inflammatory monocytes (and other secondary endpoints) with ticagrelor vs. clopidogrel argues that HRPR does not promote prothrombotic inflammation after TAVR. Furthermore, we observed only weak relationships (assessed by bivariate correlations and principal components analysis) between the markers of platelet activation and inflammation in this setting. Such compartmentalization of platelet activation and immunologic variance suggests that P2Y12 is either not an important driver of immune activation after TAVR and/or that this mechanism is adequately suppressed by the P2Y12 inhibition afforded by clopidogrel.

### Clinical and Research Implications

This study has implications for those referred for TAVR who also require percutaneous coronary intervention. Rates of major adverse cardiovascular events in this high risk subset (∼25% of the TAVR population) approach 30% within 2 years.^48^ Our data show a majority of patients experience on-clopidogrel HRPR after TAVR, and this platelet hypo-responsiveness can be circumvented by the use of ticagrelor. Increased major bleeding or adverse events between groups was not detectable in this small sample size. Larger studies powered for clinical endpoints are ultimately needed to establish whether high potency P2Y12 inhibition reduces clinical events in those undergoing TAVR who have an indication for DAPT.

The fact that greater platelet inhibition conferred by ticagrelor did not translate to reductions in immunologic biomarkers suggests that residual P2Y12 activity is not a major driver of monocyte activation in the post-TAVR setting. Instead, the association between HRPR and inflammatory monocyte expansion would suggest HRPR to be an indicator (not a cause) of innate immune activation in this setting. Additional study is needed to determine which immune processes link to adverse post-TAVR outcomes, distinguish cause from consequence/epiphenomena in this setting, and ultimately establish whether interruption of offending immunologic pathways improves intermediate term outcomes in those who undergo TAVR.

### Limitations

This study was a mechanistic design featuring deep immunophenotypic analysis on a modest sample size and should be interpreted within these constraints. Our power to detect modest differences between groups or clinical endpoints is limited. The analysis of multiple biomarkers introduces the possibility of type 1 and type 2 error. To minimize this, we prespecified co-primary endpoints on day 1, but other biomarker comparisons and analyses should be regarded as hypothesis generating. We did not specifically assess potential platelet-independent effects of ticagrelor on immune function (e.g., equilibrative nucleoside transporter 1 inhibition^49^) so inferred platelet-mediated effects may be clouded by pleotropic effects.

## Ethics Statement

The study was approved by the Institutional Research Board (IRB) at University Hospitals Cleveland Medical Center, and complied with the Declaration of Helsinki, the International Conference on Harmonization/Good Clinical Practice guidelines, and applicable local regulatory requirements. All patients provided informed consent prior to enrollment. The trial was registered at ClinicalTrials.gov (Identifier: NCT02486367). The protocol was granted an investigational new drug (IND) waiver from the United States Federal Drug Administration prior to study initiation. A data safety and monitoring board was appointed and met at quarterly enrollment intervals to review adverse events throughout the trial period. The study was an investigator-initiated study funded by AstraZeneca (Co-Principal Investigators: DAZ and DIS). The funders had no role in study design, data collection and analysis, decision to publish, or preparation of the manuscript.

## Funding

The study was an investigator-initiated study funded by 10.13039/100004325AstraZeneca (Co-Principal Investigators: D.A.Z. and D.I.S.). The funders had no role in study design, data collection and analysis, decision to publish, or preparation of the manuscript.

## Disclosure Statement

David A. Zidar reports research grant support from AstraZeneca, honoraria from GSK/Pfizer and Medtronic. Steven Filby is a consultant for Boston Scientific. Nicholas T. Funderburg is a consultant for Gilead. Mehdi Shishebhor is a consultant for Abbott Vascular, Medtronic, Terumo, Philips, and Boston Scientific. Sahil A. Parikh serves on the advisory Board of Abbott, Boston Scientific, Medtronic, Philips, Janssen, Cordis, Efemoral, and Advanced Nanotechnologies; receives research support from Abbott, Boston Scientific, surmodics, shockwave, and trireme; and is a consultant for Inari, Penumbra, Abiomed, and Terumo. Guilherme Attizzani is a consultant and advisory board member of Medtronic and Abbott Vascular. Daniel I. Simon reports honoraria from Medtronic.

## Supplementary Material

Supplementary Study Protocol
