# Supplementary material for: Platelet and Monocyte Activation After Transcatheter Aortic Valve Replacement (POTENT-TAVR): A Mechanistic Randomized Trial of Ticagrelor Versus Clopidogrel
Source: Struct Heart. 2023 Apr 28;7(4):100182. doi: 10.1016/j.shj.2023.100182 (PMC10382989; doi:10.1016/j.shj.2023.100182)
Supplement: Supplementary Study Protocol [file mmc1.docx]

**Supplementary Clinical Study Protocol**

**Title: Platelet and Monocyte Activation After Transcatheter Aortic Valve Replacement (POTENT-TAVR)**

**Subtitle:** **POTENT-TAVR: A Mechanistic Randomized Trial of Ticagrelor versus Clopidogrel**

**Table of Contents**

**1. Cover page …………………………………………… ……………………………….………2**

**2. Synopsis………………………………………………… ……………………………….…….3**

**3. Introductory Background…………………………… ………………………………..……..4**

**4. Study objectives………………………………………… ………………………………..…..6**

**5. Study design………………………………………………… ………………………….……..7**

**6. References………………………………………… ………………………………..………...12**

**1. Cover page**

**Title:** **Platelet and Monocyte Activation After Transcatheter Aortic Valve Replacement (POTENT-TAVR):** **POTENT-TAVR: A Mechanistic Randomized Trial of Ticagrelor versus Clopidogrel**

**Acronym**: POTENT_TAVR

**Test drug:** Ticagrelor

**Active Control:** Clopidogrel

**Clinical study phase:** Phase 4

**Sponsor:** AstraZeneca

**Principal Investigator:**

David A. Zidar MD, PhD

Associate Professor of Medicine

Case Western Reserve School of Medicine

Cleveland, OH 44106

Daz21@case.edu

**Compliance Statement:**

The study will be conducted in compliance with the protocol and any applicable regulatory requirements.

**Confidentiality**

The information provided in this document is strictly confidential and is intended solely for the guidance of clinical investigation. Reproduction or disclosure of this document - whether in part or in full - to parties not associated with the clinical investigation, or its use for any other purpose, without the prior written consent is not permitted.

**2. Synopsis**

**Title**: Platelet and Monocyte Activation After Transcatheter Aortic Valve Replacement (POTENT-TAVR)

**Clinical study phase**: Phase 4

**Study objective(s)**: 1. To determine whether high-potency ADP receptor blockade reduces measures of platelet activation in patients after TAVR. 2. To determine whether high-potency ADP receptor blockade mitigates the pro-thrombotic inflammatory response observed after TAVR.

**Test drug:** Ticagrelor

**Dose**: 90mg twice daily

**Route of administration**: Oral

**Duration of treatment**: The first dose of Ticagrelor will be started (180mg load) the morning of planned TAVR. Treatment will continue for 30 days.

**Active Comparator:** Clopidogrel

**Dose**: 75 mg once daily

**Route of administration**: Oral

**Duration of treatment**: The first dose of Clopidogrel will be given (300 mg load if P2Y12 naïve or 75mg if P2Y12 non-naive) the morning of planned TAVR. Treatment will continue for 30 days.

**Background treatment:** Cardiovascular medications will be continued without planned dose titration as per standard after TAVR. Low dose aspirin 81mg once daily will be initiated on the day of TAVR or continued as pre-existing therapy.

**Indication**: Aortic Stenosis and planned transcatheter aortic valve replacement (TAVR).

**Primary Endpoints**: P2Y12 platelet responsiveness (PRU) using the VerfiyNow assay at 1 day post procedure and the percentage of inflammatory (CD14+CD16+) monocytes (compared to total monocytes) 1 day after TAVR.

**Key inclusion criteria:** 1) Valvular heart disease and a clinical indication for TAVR, 2) Age of 18 years or older, 3) Capable of informed consent. 4) Planned transfemoral TAVR.

**Key exclusion criteria:** 1) Prior history of stroke, transient ischemic attack (TIA), or intracranial hemorrhage, 2) Established bleeding diathesis or thrombocytopenia (<150k/dl), 3) End-stage renal disease, 4) Severe hepatic impairment or liver cirrhosis, 5) Pregnancy, 6) Current infection, 7) History of autoimmune disease, 8) Established allergy to contrast agents, thienopyridines, aspirin, or ticagrelor, 9) History of solid organ transplantation, 10) Atrial Fibrillation, DVT, PE or other indication for long term anti-coagulation, 11) Plan for direct aortic access or trans-apical TAVR, 12) Enrollment in another clinical trial, 13) Recent (< 12 months) or active excessive bleeding.

**Type of control:** Active treatment

**Clinical Event Committee:** No

**Data Safety Monitoring Board** Yes

**Number of subjects:** A total of 60 subjects will be randomized.

**3. Introductory Background**

Transcatheter Aortic Valve Replacement (TAVR) has emerged as an important alternative to surgical aortic valve replacement. While this technology represents an important advance over medical therapy or surgical AVR in poor operative candidates, the absolute mortality rates remain high, even in the great majority in whom an optimal hemodynamic result is achieved. In the randomized literature, the majority of these patients die within two years and two thirds of these deaths are due to cardiovascular (CV) events^1^.

The mechanisms responsible for this limited survival are unclear from the clinical trials completed to date. While persistent valve disease undoubtedly plays a role in a subset of patients, particularly in patients with significant aortic regurgitation, the majority of events are due to non-valve related co-morbidities.

Our multi-disciplinary group includes members with broad expertise in cardiovascular and immunologic clinical research. The over-arching aim of our collaborative work to date is to identify shared mechanisms of inflammation and thrombosis that correlate with cardiovascular disease. Recent work from our group has focused on the prothrombotic phenotype of activated inflammatory monocytes which are preferentially expanded in both HIV and ACS^2^.

****Clopidogrel treated patients following TAVR have a 64.3% rate of platelet hyporesponsiveness (VerifyNow assay, hyporesponsiveness defined as PRU <208)^3^. In this post-TAVR population (n=28) the median PRU was 244.5 (interquartile range 164.8-290.3). In preliminary experiments, we find a profile of immune activation after TAVR characterized by the expansion of CD14+CD16+ inflammatory monocytes, a marker we and others have found correlates with adverse cardiovascular events ^2,4^. Of note, we have previously demonstrated that inflammatory monocytes express high levels of tissue factor and this feature also correlates with ACS^2^. Thus, post-TAVR patients represent a patient population in which high-potency ADP receptor blockade may provide protection against MACE.

**Figure 1.** Mechanistic hypothesis of TAVR leading to platelet activation and inflammation

***Hypothesis***

Our hypothesis is that TAVR results in at least three simultaneous CV insults: 1) the abrupt release of severely elevated left ventricular pressure into a non-compliant systemic vasculature leads to generalized endothelial cell activation, 2) the exposure of the pro-thrombotic and neo-antigenic contents of a degenerated aortic valve (known to histologically resemble atherosclerosis), and 3) the exposure of the replacement valve (bovine valve, stainless steel frame, polyester wrap). We propose that these proximate events lead to platelet activation. Given the important link between thrombosis and inflammation governed by platelet-derived mediators and leukocyte-platelet interactions, we further hypothesize that monocyte activation is mediated, at least in part, by platelet-monocyte interactions, which has been shown to induce the expansion of inflammatory monocytes^5^. Given the pro-thrombotic nature of inflammatory monocytes, we suspect a positive feedback loop may exist via the interplay of these thrombotic –inflammatory mechanisms, which may be abrogated via high potency ADP-receptor blockade (Figure 1).

If our study results indicate an improved thrombotic and immune convalescence with Ticagrelor, we would conclude that the mechanism of inflammation after TAVR is mediated in part by platelet activation regulated by the P2Y12 receptor. This would form the basis for additional study of the clinical effects of P2Y12 blockade post TAVR, powered for clinical events. Importantly, the findings of this study might also provide mechanistic proof of principle for additional study of P2Y12 blockade in other disorders that carry elevated CV risk in the setting of chronic immune activation. One such population is the vast majority of HIV patients, who achieve controlled viremia but are left with chronic immune activation and elevated CV risk^6^.

***Study Drug Background: Ticagrelor***

FDA Label: https://www.accessdata.fda.gov/drugsatfda_docs/label/2015/022433s015lbl.pdf Ticagrelor is approved by the United States Federal Drug Administration (FDA) to reduce the rate of cardiovascular death, myocardial infarction, and stroke in pateints with acute coronary syndrome or a history of myocardial infarction. Ticagrelor was studied in the PLATO trial which included patients with acute coronary syndrome, randomized to Ticagrelor or Clopidogrel for 12 months. At 12 months, a composite of death from vascular causes, myocardial infarction, or stroke (the primary endpoint) had occurred in 9.8% of patients receiving ticagrelor as compared with 11.7% of those receiving clopidogrel (hazard ratio, 0.84; 95% confidence interval [CI], 0.77 to 0.92; P<0.001). No significant difference in the rates of major bleeding was found between the ticagrelor and clopidogrel groups (11.6% and 11.2%, respectively; P=0.43), but ticagrelor was associated with a higher rate of major bleeding not related to coronary-artery bypass grafting (4.5% vs. 3.8%, P=0.03), including more instances of fatal intracranial bleeding and fewer of fatal bleeding of other types.

**4. Study objectives**

Transcatheter aortic valve replacement (TAVR) has been associated with reductions in CV morbidity and death in extreme risk/surgery inadvisable patients, but cardiovascular events rates remain unacceptably high, with mortality rates of 30% and 50% at 1 and 2 years, respectively^1,7^. Thus, even after successful treatment of aortic stenosis, this population persists with a risk of CV mortality that is 5-fold greater than cohorts studied in contemporary myocardial infarction trials such as PLATO^8^.

The mechanisms and predictors of adverse outcomes following TAVR are poorly understood. Among the most important predictor of poor outcomes is peri-procedural stroke, which is associated with a hazard ratio of 3.5 for mortality at 30 days^9^. Since 45% of cerebral-vascular accidents (CVA) occur beyond 24 hours post procedure^10^, optimizing anti-thrombotic therapies in the immediate post-procedure period may be a feasible strategy to reduce CVA following TAVR. However, the majority of major adverse cardiovascular events (MACE) following TAVR remains unexplained and thus, a better understanding of the mediators of CV risk is urgently needed in this high risk population. The role of anti-platelet therapy in this patient population is also uncertain. While dual anti-platelet therapy with aspirin and clopidogrel is typically recommended for a period of 6 months, its effectiveness in the prevention of adverse outcomes post-TAVR is unknown^11^.

In patients with cardiovascular disease, among the strongest and most consistent surrogates of poor long term outcome is clopidogrel non-responsiveness. This is typically identified in 20-30% of patients with acute coronary syndromes (ACS) and is associated with an increased risk of MACE^12-15^. Although the prevalence of clopidogrel non-responsiveness in patients with aortic stenosis has not been previously reported, preliminary data from our group indicates a high rate of clopidogrel hyporesponsiveness (64.3%) in our post-TAVR population.

Another marker of poor prognosis in patients with cardiovascular disease is elevated proportions of CD14+CD16+ inflammatory monocytes. In an unselected cardiac catheterization population, elevated CD14+CD16+ inflammatory monocytes were associated with a hazard ratio of 3.1 for future death, MI, or stoke^4^. Our studies have recently shown that these monocytes express high levels of tissue factor and are dynamically regulated in the setting of both HIV and ACS^2^. Interestingly, we will now report that TAVR is associated with a robust, inflammatory response characterized by a marked expansion of CD14+CD16+ inflammatory monocytes, evident even within 24 hours of an uncomplicated procedure.

**The central hypothesis of our proposal is that TAVR-induced injury of the diseased native aortic valve leads to platelet deposition and inflammatory cell activation that can be attenuated by the potent anti-platelet and/or pleiotropic effects of ticagrelor.**

We propose a single center, prospective randomized trial to test the following specific aims:

1. **To determine whether high-potency ADP receptor blockade reduces measures of platelet activation in patients after TAVR.**
2. **To determine whether high-potency ADP receptor blockade mitigates the pro-thrombotic inflammatory response observed after TAVR.**

The co-primary study endpoints will be 1) platelet responsiveness as measured by the VerifyNow assay (platelet reactivity units, PRU) and 2) the proportion of inflammatory monocytes by polychromatic flow cytometry 1 day after TAVR. Secondary endpoints will include other measures of thrombotic and immunologic activation.

**5. Study design**

This is a single center, prospective, randomized, open label trial with blinded endpoint evaluation.

**TABLE 1. Inclusion/Exclusion Criteria**

**Inclusion criteria:**

1) Valvular heart disease and a clinical indication for TAVR,

2) Age of 18 years or older,

3) Capable of informed consent.

4) Planned transfemoral TAVR.

**Exclusion criteria:**

1) Prior history of stroke, transient ischemic attack (TIA), or intracranial hemorrhage,

2) Established bleeding diathesis or thrombocytopenia (<150k/dl),

3) End-stage renal disease,

4) Severe hepatic impairment or liver cirrhosis,

5) Pregnancy,

6) Current infection,

7) History of autoimmune disease,

8) Established allergy to contrast agents, thienopyridines, aspirin, or ticagrelor,

9) History of solid organ transplantation,

10) Atrial Fibrillation, DVT, PE or other indication for long term anti-coagulation,

11) Plan for direct aortic access or trans-apical TAVR,

12) Enrollment in another clinical trial,

13) Recent (< 12 months) or active excessive bleeding.

**5.1 Screening, entry, and randomization:** Patients will be screened for inclusion/exclusion criteria as part of their pre-TAVR clinical evaluation (See Table 1). We plan to screen 150 patients per year, of whom 100 are expected to go on to TAVR. We will continue enrollment until 60 patients are randomized. All patients will be required to sign informed consent prior to planned TAVR. The scheme for a participant’s movement through the trial is shown in figure 2.

Patients will be randomized in a 1:1 fashion (n=60) to either continue Clopidogrel (300mg load, 75mg daily) or begin Ticagrelor. Patients will receive a 180mg load followed by 90mg twice daily for one month. This will be an open label design given the different frequency of dosing. Decisions regarding the discontinuance of dual anti-platelet therapy after randomization will be at the discretion of the treating physician.

Patients will be seen at 30 days post TAVR per routine clinical practice. At this time, the final blood draw will be obtained. The occurrence of MACE and bleeding events will be determined by history and corroborated by examining relevant medical records. Patients will revert to clopidogrel therapy at this time and the duration of dual anti-platelet therapy will be left to the discretion of the treating physician.

**5.2 Blood Biomarker Procurement**:

Patients will receive a baseline blood draw prior to TAVR. Patients will then have blood draws at 24 hours, 7 days, and 30 days post procedure. Patients will be seen in followup according routine clinical practice at 7 and 30 days. All blood samples will be obtained using a 30 mL syringe which is then transferred into tubes containing 3.2% sodium citrate (blue top) or K2-EDTA (purple top). Aliquots of whole blood will be used for platelet responsiveness studies, monocyte flow cytometry, leukocyte-platelet interaction, and T cell activation studies. Plasma and Peripheral blood mononuclear cells (PBMC) will be cryopreserved at -80 and -150 degrees, respectively. Plasma based assays will be batched and performed at intervals using freshly thawed cryopreserved samples.

**5.3 Study Endpoints and Statistical Plan**

All endpoints will be determined according to an intention to treat analysis. The primary thrombotic endpoint will be platelet responsiveness (PRU) using the VerfiyNow assay at 1 day post procedure. Platelet reactivity in the Clopidogrel and Ticagrelor groups with be compared using an independent samples t-test (or non-parametric test as appropriate), with significance declared for p-values <0.025. The primary inflammatory endpoint is the percentage of inflammatory monocytes (compared to total monocytes) as a continuous variable 1 day after TAVR. The percentage of inflammatory monocytes in the Clopidogrel and Ticagrelor groups with be compared using an independent samples t-test (or non-parametric test as appropriate), with significance declared for p-values <0.025. The percentage of inflammatory monocytes will also be compared over multiple time points to determine the trajectory and duration of the immune response, to guide duration of treatment in future studies. The values at baseline, 1 day, and 7 day will be compared within group using exploratory longitudinal mixed models to determine the kinetics of convalescence and between groups to establish whether treatment effects exist. Models that include the 30-day values also will be fit. Time period will be considered both as a continuous covariate (in days), as well as a categorical variable. Inferential interest will focus on treatment by time interaction. These models will also be used to explore the relationship between the level of P2Y12 inhibition and the reduction in inflammatory monocytes between treatments. This relationship could be different given that Ticagrelor is not only a more potent P2Y12 antagonist as compared with clopidogrel but may also have a unique dual mode of action; P2Y12 antagonism complemented with adenosine uptake inhibition.

**Figure 2.** Trial Design Schematic

Secondary measures of thrombotic and immune status will be crucial for the determination of mechanism and to guide future studies. These will include: platelet-leukocyte aggregates, platelet P-selectin and activated GPIIb/IIIa expression, markers of T-cell activation (i.e., HLA-DR/CD38 co-expression), and plasma markers of inflammation (CRP, IL-6, TNF-a, IL-6, sCD14, CD40L, oxidized LDL) and thrombosis (thrombin-antithrombin complexes, F1.2, soluble P-selectin, S100A8/A9, D-dimers). These secondary measures will be analyzed as continuous variables at baseline, 1 day, 7 days, and 30 days. PBMC will be isolated and will be used to assess T cell activation as continuous variables using the percentage of HLA-DR+/CD38+ CD4 and CD8 T-cells. These values at the specified time points will also be compared within group using longitudinal mixed models to determine their kinetics and between groups to establish whether a treatment by time interactions exists.

**5.4 Time point/frame of measurement for primary variable(s)**

The primary endpoints will be assessed 1 day after study drug initiation and TAVR. Measurements taken at 7 and 30 days after TAVR will be considered secondary/exploratory.

**5.5. Study variable(s)**

*5.5.a Clinical/Demographic Variables*: Demographic and clinical variables will be captured by interview and electronic chart review at study entry.

*5.5.b P2Y12 Platelet Reactivity*: Platelet reactivity to adenosine diphosphate (ADP) will be measured as platelet reactivity units (PRU) using the VerifyNow assay (Accriva Diagnostics, San Diego, California) using whole blood collected in a device- compatible citrated tube.

*5.5.c Whole Blood Flow Cytometric Analysis of Patient Samples:*

| **Monocyte Panel A** | | | | | |
| --- | --- | --- | --- | --- | --- |
| **Target** | **Fluorochrome** | **Vendor** | **Catalog Number** | **Clone** | **Dilution** |
| CD14 | Pacific Blue | BD Bioscience | 558121 | M5E2 | 1:50 |
| Tissue Factor | FITC | Biomedica Diagnostics | 4507CJ | 23C11 | 1:50 |
| CD16 | PE | BD Bioscience | 555407 | 3G8 | 1:50 |
| CD62p | Pe-Cy5 | BD Bioscience | 551142 | AK-4 | 1:50 |
| CD69 | Pe-Cy7 | BD Bioscience | 335792 | L78 | 1:50 |
| CX3CR1 | APC | BioLegend | 341610 | 29A-1 | 1:50 |
| HLA-DR | APC-Cy7 | BD Bioscience | 335796 | L243 | 1:50 |
|  | | | | | |
| **Monocyte Panel B** | | | | | |
| **Target** | **Fluorochrome** | **Source / Vendor** | **Catalog Number** | **Clone** | **Dilution** |
| CD14 | Pacific Blue | BD Bioscience | 340507 | M5E2 | 1:50 |
| GP IIb/IIIa | FITC | BD Bioscience | 347363 | PAC-1 | 1:50 |
| MRP8/14 | PE | Novus Biologicals | NBP2-25269PE | 48M7C7 | 1:250 |
| CD41 | Pe/Cy5 | BioLegend | 303708 | HIP8 | 1:50 |
| CD16 | Pe-Cy7 | BD Bioscience | 557744 | 3G8 | 1:50 |
| CX3CR1 | APC | BioLegend | 341610 | 29A-1 | 1:50 |
| CD62l | APC-eFluor780 | eBioscience | 47-0629-42 | DREG-56 | 1:50 |

Blood samples will be lysed using BD FACS lysing solution (BD, Franklin Lakes, NJ), washed, and stained as Table 2. Compensation will be performed using standard compensation beads (BD Biosciences) and appropriate isotype control antibodies. Flow cytometry will be performed using a MACSQUANT analyzer. Monocyte phenotypes (classical, non-classical inflammatory CD14+CD16+, non-classical patrolling CD14lowCD16++) will be analyzed by whole blood flow cytometry using antibodies to CD14 and CD16 as we have previously described^16^. Specifically, the % inflammatory monocytes is the proportion of inflammatory monocyte to the sum of classical, inflammatory and patrolling monocytes. The definition of CD16 positive versus negative gating will be based upon a pan-isotype panel. Final values for % inflammatory monocytes will be the mean of the duplicates obtained using panel A and panel B. Monocyte-CD62P+ platelet aggregation will be analyzed as the mean fluorescence intensity (MFI) of P-selection (Monocyte Panel A) and Monocyte- GP IIb/IIIa+ platelet aggregation will be analyzed as the percentage of monocytes which exhibit positive staining for activated IIb/IIIa (PAC-1 antibody- Monocyte Panel B), with total monocytes as the denominator.

*5.5.d Blood Collection and Isolation of Peripheral Blood Mononuclear Cells and Plasma*: 30 mL blood was collected in tubes containing ethylenediaminetetraacetic acid (EDTA; BD Vacutainer, Franklin Lakes, NJ) and 2 mL in tubes containing 3.2% sodium citrate (BD Vacutainer, Franklin Lakes, NJ). Samples were then maintained at room temperature until processing. Plasma was isolated from the EDTA blood through centrifugation and preserved at -80 degrees Celsius. Peripheral blood mononuclear cells (PBMC) were isolated by Ficoll-Paque Plus (Sigma, St. Louis, MO) density gradient centrifugation, washed, re-suspended in 90% fetal bovine serum (FBS; Gemini, West Sacramento, CA) and 10% dimethylsulfoxide (DMSO; Sigma, St. Louis, MO), and cryopreserved at -150 degrees F.

*5.5.e Plasma Cytokine and Immune Biomarkers*: Plasma based assays are done using commercially available ELISA and cytokine bead array technology (BD Biosciences).

**5.6 Power Calculation and Sample Size Estimate**

Our power calculation is based upon the inflammatory co-primary endpoint, using our preliminary data. In preliminary studies of 25 patients undergoing TAVR, the mean proportion of inflammatory monocytes 1 days after TAVR was 20.0% with a standard deviation of 5.0 %. Thus, a total of 60 patients, randomized 30:30 would be expected to have 80% power to detect an absolute effect of 4% (i.e. ticagrelor treated patients having 16 % percent inflammatory monocytes), using a two sided t-test with Type I error of 0.025. Note that we are using Bonferonni correction, to account for two primary outcomes. We expect dropout of randomized patients to be less than 10% prior to the 1 day blood draw. All randomized patients will be analyzed using an intention to treat analysis. We will assess the missing completely at random assumption by comparing results with analyses derived by assuming that the data is missing at random (MAR), and use of multiple imputation approaches.

**5.7 Interruption or Discontinuation of treatment**

Neither erroneous randomization of subject in the presence of exclusion criteria or non-fulfilment

of inclusion criteria nor a post-randomization change in health status that results in

the subject meeting one or more of the exclusion criteria should automatically lead to the

interruption or permanent discontinuation of the assigned therapies, unless continuing the

medications places the subject at undue risk as determined by the investigator. Such

situations though very rare, may occur and should be handled on a case-by-case basis. Compliance with study drug medication will be assessed at day 1, 7, and 30. Drug supplies for each subject will be inventoried and reconciled throughout the study. Any discrepancies between actual and expected amount of study medication will be discussed with the subject at the time of the visit, and any explanation will be documented in the source records and captured in the eCRF. Subjects will return empty study drug containers and unused study drug visits. Study assigned therapies may be temporarily discontinued at the discretion of the treating physician. Subjects can resume the assigned therapy as soon as the treating physician considers it safe to do so. The reason for discontinuation of the assigned therapy will be recorded in the eCRF and in the subject's medical records. The decision to restart or permanently withdraw study medication is at the discretion of the investigator.

**5.8 Treatment assignment**

Randomization will be performed via seal envelop system. Opaque envelopes (60) will be filled with a sheet of paper in which Ticagrelor (30) or Clopidogrel (30) is written and envelops are to be opened after informed consent is received and on or before the start of the TAVR such that study drug initiation occurs prior to TAVR. Randomization envelops are stored securely at the Clinical trials office at University Hospitals (UH).

**5.9 Blinding**

This trial is an open label trial and patients are not blinded. The laboratory staff will receive study samples containing patient study identification numbers but without any identifiers with regard to treatment assignment. Similarly, the analysis of flow cytometric data will be done by laboratory staff blinded to treatment assignment.

**5.10. Drug logistics and accountability**

Ticagrelor and Clopidogrel will be stored at the investigational pharmacy at University Hospitals Cleveland Medical Center in accordance with GCP and GMP requirements and according to the product label. Receipt, distribution, return, and destruction (if any) of study medications will be properly documented.

**5.11 Prior and concomitant therapy**

All ongoing medications at screening and used during the study (prescription and over-the counter), including adjunct therapy or medical devices, will be recorded in the appropriate

section of the eCRF throughout the study. Use of ASA and clopidogrel or any other P2Y12

inhibitor prior and during TAVR procedure should also be recorded. It is advised that the appropriate local and national guideline recommendations are followed for any concomitant medications. Concomitant medication will be evaluated when considering AE causal relationship.

**5.12 End of study**

The end of the study as a whole will be reached 2 years after the last subject has

been enrolled.

**References**

1. Makkar RR, Fontana GP, Jilaihawi H, et al. Transcatheter Aortic-Valve Replacement for Inoperable Severe Aortic Stenosis. *New England Journal of Medicine.* 2012;366(18):1696-1704.

2. Funderburg NT, Zidar DA, Shive C, et al. Shared monocyte subset phenotypes in HIV-1 infection and in uninfected subjects with acute coronary syndrome. *Blood.* 2012;120(23):4599-4608.

3. Elad Asher PA, Rebecca A. Brauch, Ka Chun Alan Chan, Marco Costa, Tom Lassar, Kehllee Popovich, Daniel Simon. Characteristics and outcomes of clopidogrel responder and hypo-responder patients post transcatheter aortic vavle replacement. *JACC.* 2013;62(18 Supplement B):B221-222.

4. Rogacev KS, Cremers B, Zawada AM, et al. CD14++CD16+ Monocytes Independently Predict Cardiovascular Events: A Cohort Study of 951 Patients Referred for Elective Coronary Angiography. *Journal of the American College of Cardiology.* 2012;60(16):1512-1520.

5. Passacquale G, Vamadevan P, Pereira L, Hamid C, Corrigall V, Ferro A. Monocyte-Platelet Interaction Induces a Pro-Inflammatory Phenotype in Circulating Monocytes. *PLoS ONE.* 2011;6(10):e25595.

6. Triant VA. HIV Infection and Coronary Heart Disease: An Intersection of Epidemics. *Journal of Infectious Diseases.* 2012;205(suppl 3):S355-S361.

7. Kodali SK, Williams MR, Smith CR, et al. Two-Year Outcomes after Transcatheter or Surgical Aortic-Valve Replacement. *New England Journal of Medicine.* 2012;366(18):1686-1695.

8. Wallentin L, Becker RC, Budaj A, et al. Ticagrelor versus Clopidogrel in Patients with Acute Coronary Syndromes. *New England Journal of Medicine.* 2009;361(11):1045-1057.

9. Eggebrecht H, Schmermund A, Voigtl T, Kahlert P, Erbel R, Mehta RH. Risk of stroke after transcatheter aortic valve implantation (TAVI): a meta-analysis of 10,037 published patients. *EuroIntervention.* 2012;8(1):129-138.

10. Nombela-Franco L, Webb JG, de Jaegere PP, et al. Timing, Predictive Factors, and Prognostic Value of Cerebrovascular Events in a Large Cohort of Patients Undergoing Transcatheter Aortic Valve Implantation. *Circulation.* 2012;126(25):3041-3053.

11. Rodés-Cabau J, Dauerman HL, Cohen MG, et al. Antithrombotic Treatment in Transcatheter Aortic Valve ImplantationInsights for Cerebrovascular and Bleeding Events. *Journal of the American College of Cardiology.* 2013;62(25):2349-2359.

12. Brar SS, ten Berg J, Marcucci R, et al. Impact of Platelet Reactivity on Clinical Outcomes After Percutaneous Coronary Intervention: A Collaborative Meta-Analysis of Individual Participant Data. *Journal of the American College of Cardiology.* 2011;58(19):1945-1954.

13. Sofi F, Marcucci R, Gori AM, Giusti B, Abbate R, Gensini GF. Clopidogrel non-responsiveness and risk of cardiovascular morbidity. An updated meta-analysis. *Thrombosis and Haemostasis.* 2010;103(4):841-848.

14. Combescure C, Fontana P, Mallouk N, et al. Clinical implications of clopidogrel non-response in cardiovascular patients: a systematic review and meta-analysis. *Journal of Thrombosis and Haemostasis.* 2010;8(5):923-933.

15. Aradi D, Komócsi A, Vorobcsuk A, et al. Prognostic significance of high on-clopidogrel platelet reactivity after percutaneous coronary intervention: Systematic review and meta-analysis. *American Heart Journal.* 2010;160(3):543-551.

16. Zidar DA, Juchnowski S, Ferrari B, et al. Oxidized LDL Levels Are Increased in HIV Infection and May Drive Monocyte Activation. *JAIDS Journal of Acquired Immune Deficiency Syndromes.* 2015;69(2):154-160.
