# Supplementary material for: Platelet and Monocyte Activation After Transcatheter Aortic Valve Replacement (POTENT-TAVR): A Mechanistic Randomized Trial of Ticagrelor Versus Clopidogrel
Source: Struct Heart. 2023 Apr 28;7(4):100182. doi: 10.1016/j.shj.2023.100182 (PMC10382989; doi:10.1016/j.shj.2023.100182)
Supplement: Supplemental Tables 1-3 to Figures 1- — 5 [file mmc2.docx]

***Supplemental Methods and Results***

**Supplemental Figure 1.** CONSORT diagram of enrollment, followup and analysis of participants.


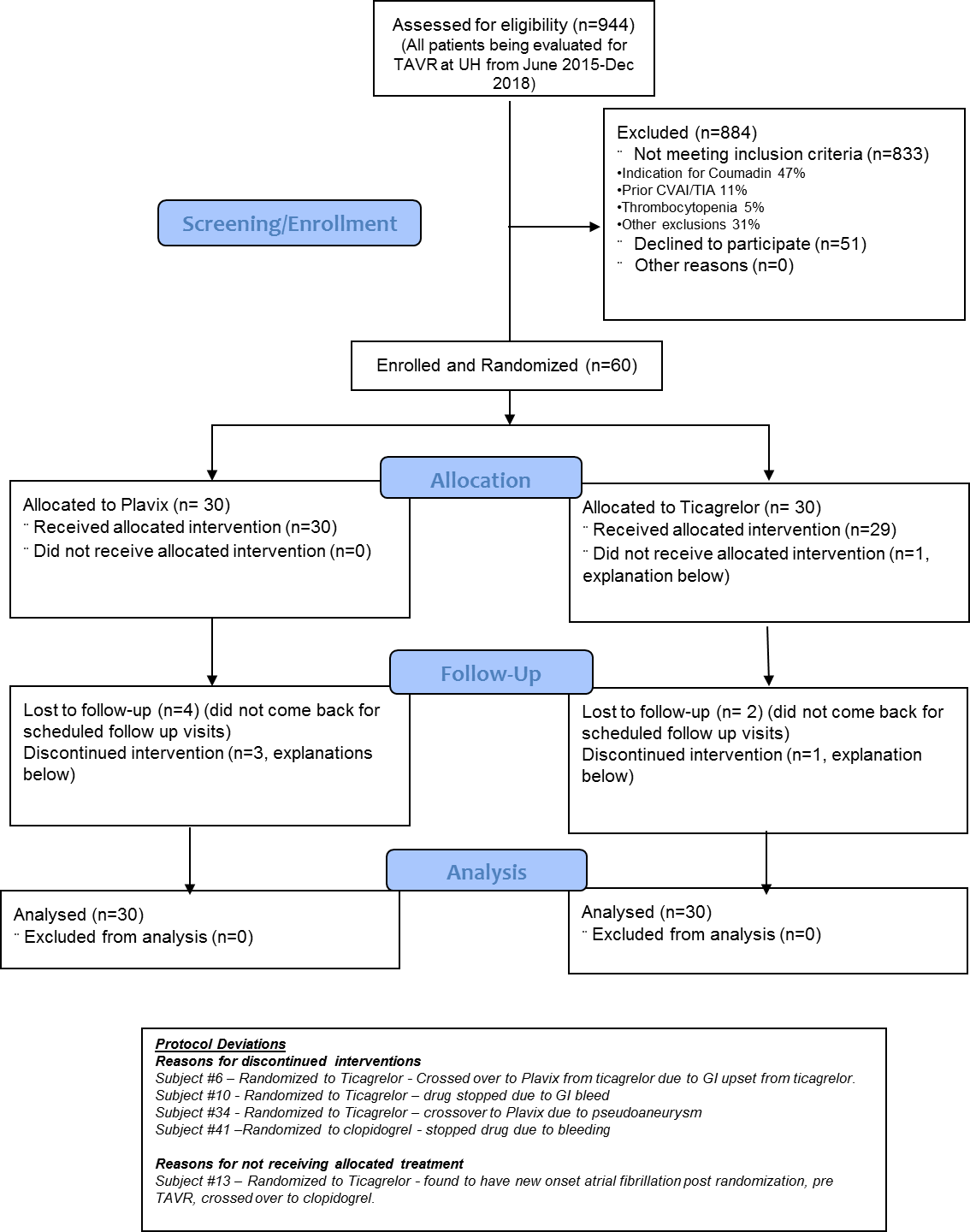


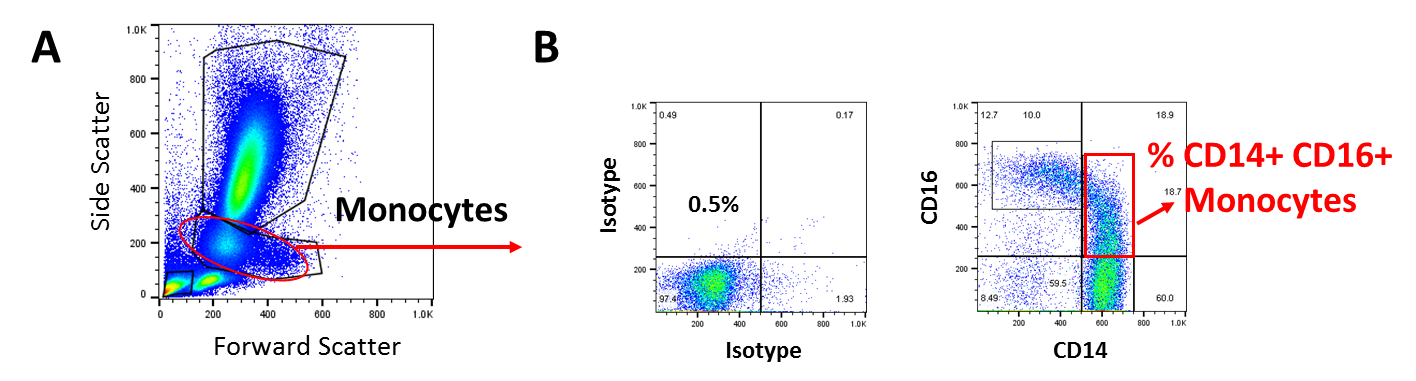


**Supplemental Figure 2. Flow Cytometry Gating Strategy for the Determination of Inflammatory Monocyte Proportions.** Fresh whole blood was analyzed by multicolor flow cytometry. 300 uL was incubated in red cell lysis buffer, washed and re-suspended in 300uL phosphate buffered saline and 100uL analyzed on a MACSQUANT analyzer. Doublets were excluded by selecting cells with uniform forward scatter area versus height. Monocytes were first selected and gated based upon intermediate forward scatter versus side scatter (A). These monocytes were then analyzed for CD14 and CD16 intensity as shown (B). The gating of inflammatory (CD14+ CD16+) monocytes was standardized using the isotype stained tube wherein the vertical set point defining CD16+ versus CD16- was based on 0.5% false positive cells in isotype stained tubes. The inflammatory monocyte endpoint was then computed as the percentage of cells within the CD14+CD16+ gate divided by the sum of cells in the CD14+ CD16- gate, CD14+ CD16+ gate and CD14dim CD16++ gate.


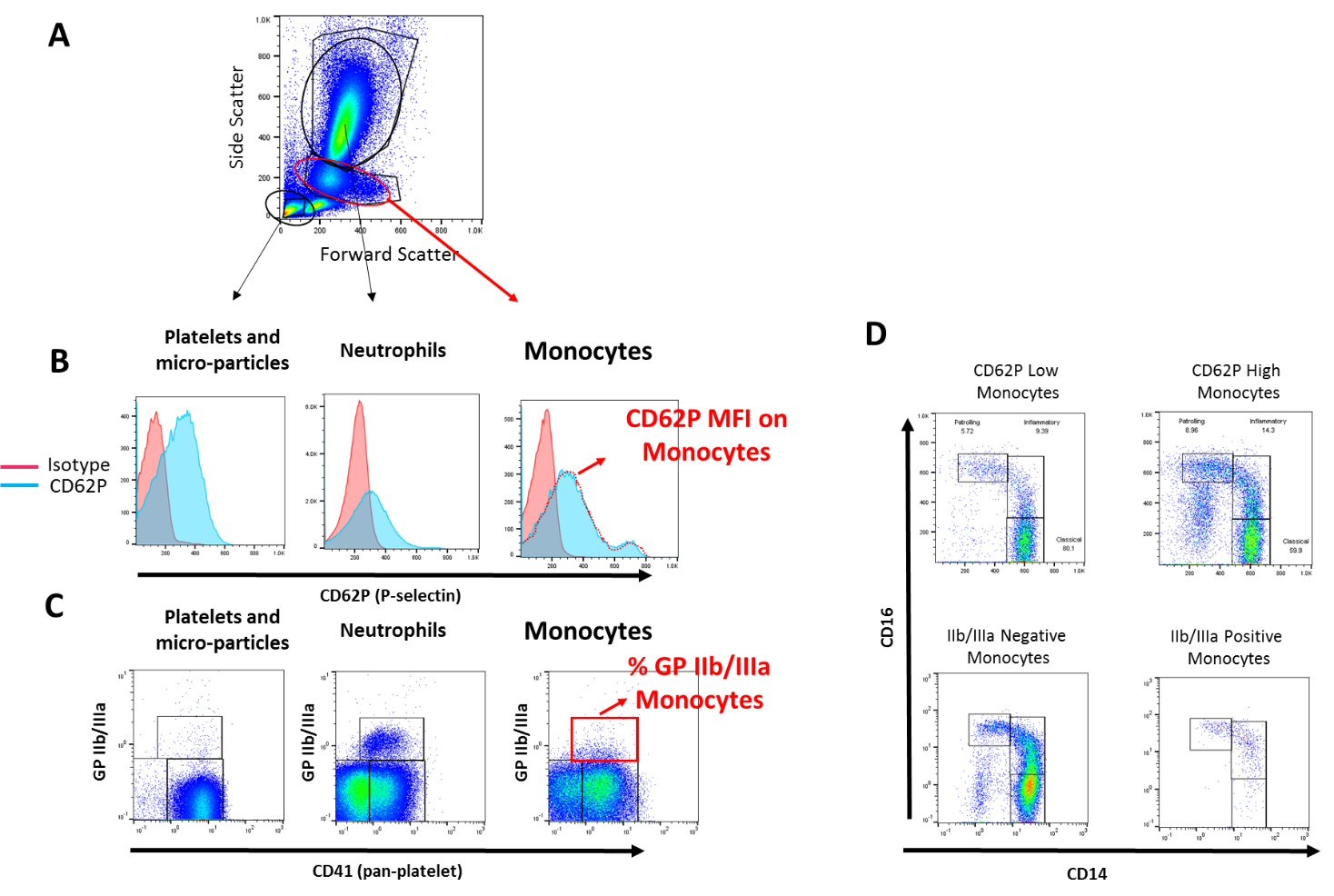


**Supplemental Figure 3. Flow Cytometry Gating Strategy for the Determination of Platelet-Monocyte Aggregates.** Fresh whole blood was analyzed by multicolor flow cytometry. 300 uL was incubated in red cell lysis buffer, washed and re-suspended in 300uL phosphate buffered saline and 100uL analyzed on a MACSQUANT analyzer. Doublets were excluded by selecting cells with uniform forward scatter area versus height. **(A)** Monocytes were first selected and gated based upon intermediate forward scatter versus side scatter. **(B)** These monocytes were then analyzed for CD62P intensity as shown and reported as the mean fluorescence intensity (MFI). **(C)** Monocytes as were also analyzed for expression of activated glycoprotein (GP) IIb/IIIa in conjunction with CD41 (a pan platelet marker). Gating for GP IIb/IIIa positivity was based upon Neutrophil staining, where it identifies a distinct population. GP IIb/IIIa positivity is reported at the percentage of cells in this gates with all monocytes gated in panel A as the denominator.  **(D)** In general, MPA-positive monocytes (when back-gated into a CD14 and CD16 analysis) were enriched for non-classical monocytes consistent with a more activated phenotype.

**Supplemental Table 1. Predictors of Major Bleeding**

**
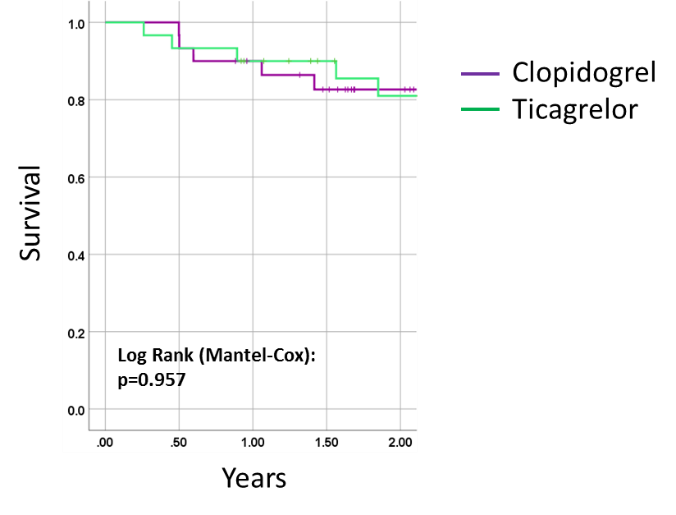
**

**Supplemental Figure 4: Survival After TAVR According to Treatment Assignment**. Kaplan-Meier curves depict overall survival after TAVR for those treated with clopidogrel (purple) or ticagrelor (green). (Log rank Mantel-Cox).

**Supplemental Table 2. Summary of Biomarker Comparisons**

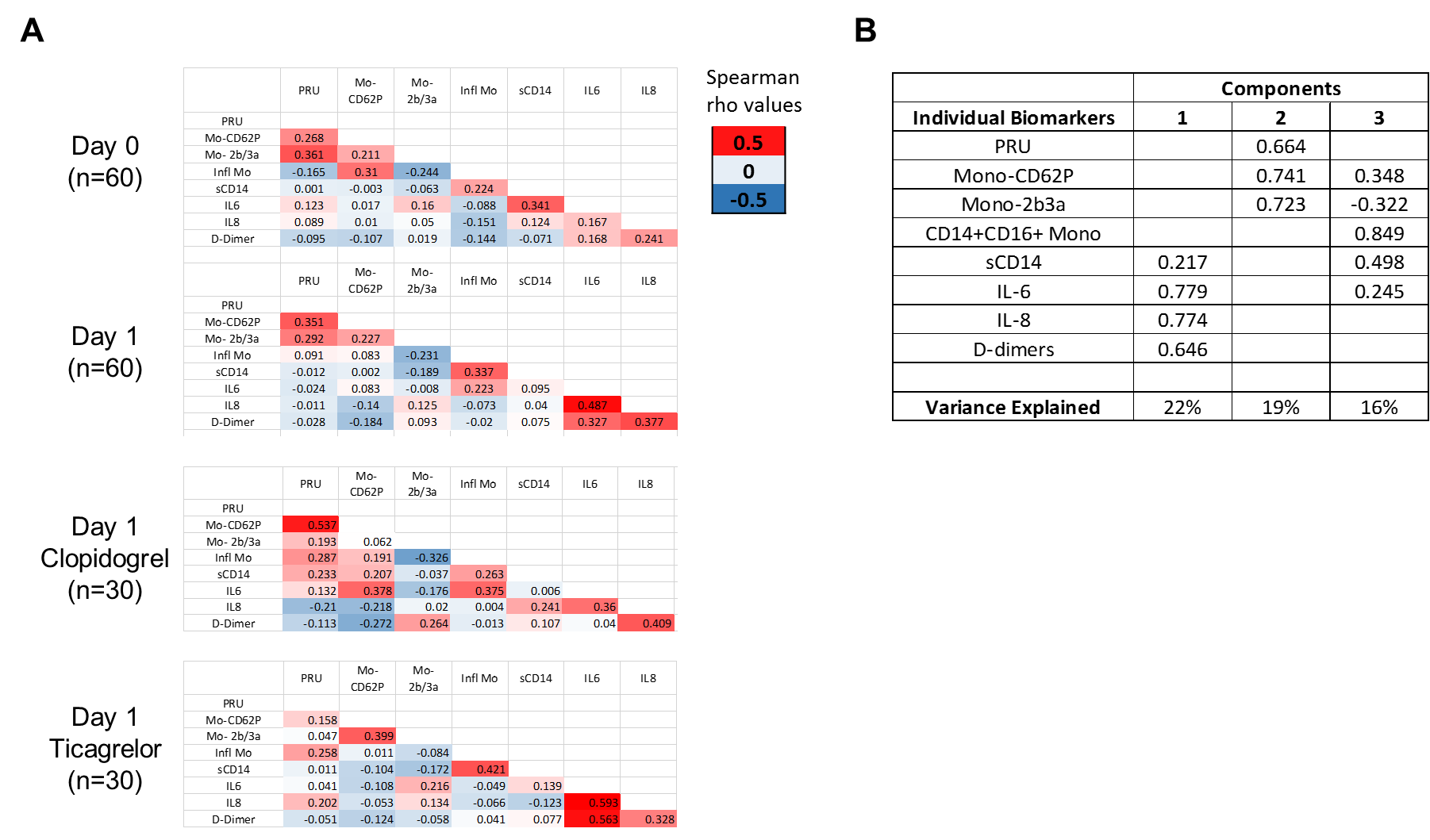


**Supplemental Figure 5: Bivariate and Multivariate Relationships among Inflammatory and Thrombotic Measures**. (**A**) Bivariate correlations for platelet P2Y12 inhibition (PRU), monocyte-platelet CD62P aggregates (mo-CD62P), monocyte-platelet glycoprotein IIb/IIIa aggregates (Mo-2b/3a), Inflammatory/CD14+CD16+ monocyte proportions (Infl Mo), soluble CD14 (sCD14), interleukin (IL)-6, IL-8, and D-dimers at day 0 and day 1 after study drug initiation/TAVR are shown. Spearman’s rho values and heat map colors represent the strength of positive (red) or inverse (blue) associations among these various biomarkers. (**B**) Standardized biomarkers from days 0,1, 7, and 30 were used for principal components analysis. The rotated component matrix table shows the contributions of individual biomarkers (values < 0.2 suppressed) to the 3 components with eigenvalues greater than 1.

**Supplemental Table 3. Predictors of High Residual Platelet Reactivity with the Clopidogrel Arm**
